# Improvement in Skin Penetration Capacity of Linalool by Using Microemulsion as a Delivery Carrier: Formulation Optimization and In Vitro Evaluation

**DOI:** 10.3390/pharmaceutics15051446

**Published:** 2023-05-09

**Authors:** Ming-Jun Tsai, Wen-Yu Chang, I-Hui Chiu, I-Ling Lin, Pao-Chu Wu

**Affiliations:** 1 School of Medicine, College of Medicine, China Medical University, Taichung 404, Taiwan; 2 Department of Neurology, China Medical University Hospital, Taichung 404, Taiwan; 3 Department of Neurology, An-Nan Hospital, China Medical University, Tainan 709, Taiwan; 4 School of Pharmacy, Kaohsiung Medical University, Kaohsiung 807, Taiwan; andy200000746@gmail.com (W.-Y.C.); u112830001@kmu.edu.tw (I.-H.C.); 5 Department of Medicine Laboratory Science and Biotechnology, College of Health Science, Kaohsiung Medical University, Kaohsiung 807, Taiwan; linili@kmu.edu.tw; 6 Department of Laboratory Medicine, Kaohsiung Medical University Hospital, Kaohsiung 807, Taiwan; 7 Department of Medical Research, Kaohsiung Medical University Hospital, Kaohsiung 807, Taiwan; 8 Drug Development and Value Creation Research Center, Kaohsiung Medical University, Kaohsiung 807, Taiwan

**Keywords:** permeation capacity, topical application, stability, response surface methodology, mixture design

## Abstract

Linalool is an aromatic oil with analgesic, anti-inflammatory and anti-UVB-induced skin damage effects. The aim of this study was to develop a linalool-loaded microemulsion formulation for topical application. In order to quickly obtain an optimal drug-loaded formulation, statistical tools of the response surface methodology and a mixed experimental design with four independent variables of oil (X_1_), mixed surfactant (X_2_), cosurfactant (X_3_) and water (X_4_) were used to design a series of model formulations in order to analyze the effect of the composition on the characteristics and permeation capacity of linalool-loaded microemulsion formulations and to obtain an appropriate drug-loaded formulation. The results showed that the droplet size, viscosity and penetration capacity of linalool-loaded formulations were significantly affected by formulation component proportions. The skin deposition amount of the drug and flux of such formulations expressively increased about 6.1-fold and 6.5-fold, respectively, when compared to the control group (5% linalool dissolved in ethanol). After 3 months of storage, the physicochemical characteristics and drug level did not show a significant change. The linalool formulation-treated rat skin showed non-significant irritation compared to skin treatments in the distilled-water-treated group. The results showed that specific microemulsion applications might be considered as potential drug delivery carriers for essential oil topical application.

## 1. Introduction

Linalool (linalyl alcohol, C_10_H_18_O, molecular weight (MW) of 154.25, 3,7-dimethylocta-1,6-dien-3-ol, water solubility of 1.589 mg/mL) is a tertiary alcohol derived from terpenes and is the main volatile component in various aromatic plant essential oils. It is a natural product found in Nepeta nepetella, Teucrium montanum and other organisms. It is a colorless-to-pale yellow liquid with an odor similar to bergamot oil and French lavender. Linalool can soluble in fixed oils, propylene glycol, alcohol and ether, and insoluble in glycerol and water. It has a viscosity of 4.465 mPa s at 25 °C (dynamic). Linalool is often used as an ingredient in various products such as perfumes, cosmetics and hygiene products [1,2,3,4].

Previous studies have reported that linalool has a wide range of physiological effects on humans including an anti-psoriatic effect, protects against the oxidative damage of UVB radiation of human skin cells by modulated NF-κB signaling and MAPK in HDFa cells, induces apoptosis in human leukemia cells, possesses sedative, anxiolytic and anticonvulsant proprieties by modulating the glutamate and GABA neurotransmitter systems, controls pain by potentiation with GABA- and glycine-induced responses and alleviates the side effects as well as tolerance to opioids (morphine) [2,3,5,6,7,8,9,10,11,12]. Unfortunately, its application is limited due to its lipophilicity (1590 mg/L in water at 25 °C), poor oral bioavailability and short half-life (about 44.72 min) [13]. Transdermal delivery systems are an attractive alternative administration to oral delivery because they can offer many advantages, including the avoidance of gastrointestinal irritations, a decreased first-pass effect, sustained dosing to provide a stable plasma pattern, is less invasive and reveals enhanced patient compliance, and so on [14,15]; moreover, the MW of linalool is less than 500, indicating that it is a suitable candidate active pharmaceutical ingredient for a transdermal delivery system [14,15]. 

Microemulsion systems (MEs) are optically isotropic and thermodynamically stable colloidal systems composed of both oily and aqueous phases, surfactants, as well as a short-chain alcohol as a cosurfactant having an average globular size in the range of 10–200 nm [16]. Due to the presence of surfactants and cosurfactants, MEs can be readily formed spontaneously and can enhance the solubility of poorly soluble active pharmaceutical ingredients in pharmaceutical formulations. As numerous studies have demonstrated the potential of such microemulsion systems in improving drug delivery through dermal and transdermal administration [17,18,19,20,21,22], microemulsions were used as drug delivery carriers to design linalool-loaded microemulsion formulations for topical applications in this study. 

Response surface methodology [20,21,22,23,24] is a dominant statistical skill to optimize pharmaceutical formulations, to elucidate the relationship between independent factors such as the type and amount of pharmaceutical excipients and the conditions of manufacturing processes and expected responses while also shortening the development time period of pharmaceutical formulations by running minimum trials. It is a significantly more efficient and cost-effective approach than the traditional pharmaceutical products development method, which has been widely used in both academic and industrial fields. The process of the response surface method includes two parts: (1) Design systematic drug formulations by using a statistical factor design such as mixed design, central combination design, etc., to minimize the number of trials and challenge the reliability of the response surface model. (2) Explore the relationship between several explanatory and response variables by using the polynomial equation regression method to realize the effect of independent variables on responses, and then to obtain the optimal formulations or conditions with target goals. Here, the aim of this study was to systematically inspect the influence of the formulation components (amount of linalool, mix surfactant and cosurfactant and distilled water) on the physicochemical properties (average globule size and viscosity) of designed formulations and the penetration capacity of linalool-loaded formulations through SD rat skin, and to obtain an optimal drug-loaded formulation by using the response surface methodology with a four-variable mixture design. Additionally, the stability of a linalool-loaded microemulsion formulation and skin irritation after 24 h of application were also examined to confirm the utility of the designed formulation.

## 2. Materials and Methods

### 2.1. Materials and Animals

Linalool, diazepam and paraformaldehyde were acquired from Sigma-Aldrich (St. Louis, MO, USA). Span 20 (sorbitan monolaurate, HLB = 8.6) was purchased from Tokyo Chemical Industry (Tokyo, Japan). Tween 40 (polyoxyethylene sorbitan monopalmitate, HLB = 15.6) was acquired from Showa Corporation (Tokyo, Japan). 1,3-propanediol was purchased from Acros organic (Geel, Belgium). All other chemicals and solvents were of analytical reagent grade. 

### 2.2. Pseudoternary Phase Diagram Construction

Construct a pseudoternary phase diagram using the water titration method [16]. Linalool (oil phase) and Tween 40/Span 20 blended surfactant as well as co-surfactant in a ratio of 1:9 to 9:1. Add distilled water dropwise to the mixture of oil, surfactant and cosurfactant in a certain weight ratio, and titrate with vortex vibration. After settling, it was judged visually whether the final mixture was a multiphase microemulsion or a two-phase mixture. Turbidity was also considered an indicator of phase separation.

### 2.3. Linalool-Loaded Formulations Preparations

A four-variable mixture design was used to arrange a series of model formulations. The linalool (X_1_, 1~5%), mixture surfactant of Tween40/Span20 (X_2_, 15~25%), 1,3-propanodiol (X_3_, 15~30%) and distilled water (X_4_, 40~60%) were set as the independent variables, respectively. The highest proportion of distilled water was used to enhance the hydration of the stratum corneum and accelerate the penetration of drug. Design-Expert software (version 9.0.0, Stat-Ease, Inc., Minneapolis, MN, USA) selected a set of candidate points, and the composition of these model linalool-loaded microemulsion formulations was shown in Table 1.

Mix the Tween 40/Span 20 Mix Surfactant well. In an amber glass bottle, place accurately weighed linalool, Tween 40/Span 20 mixture and 1,3-propanediol, vortexed for 1 min to mix well. Then, add distilled water, vortexed and mixed well to obtain a clear and transparent mixture, and finally store these model linalool-loaded microemulsion formulations at room temperature for future use.

### 2.4. Physicochemical Properties Determination 

A cone-plate of viscometer (Model LVDV-II, Brookfield, NY, USA) was used to measure the viscosities of linalool-loaded microemulsion formulations. Load 0.5 mL of sample into the cone-plate, heat the cone-plate with a thermostatic pump and keep it at 37 °C for 3 min. The rotational speed was set at 100.0 rpm, and the viscosity measurement was recorded after 30 s.

The Zetasizer analyzer (Malvern, 3000HSA, Malvern, UK) was used to determine the average droplet size and polydispersity index (PI) of linalool-loaded microemulsion formulations. The conditions determined included a wavelength of 658 nm, a scan angle of 90° and a temperature of 25 °C. Place 3 mL of the sample into a standard quartz cuvette and then into a scattering chamber to determine the mean droplet size and PI. Each test sample was measured in triplicate and the average value is given.

### 2.5. In Vitro Permeation Study 

All animal experiments were performed in accordance with the approval obtained from Kaohsiung Medical University, Institutional Animal Ethics Committee (approval number 104131), and their guidelines were followed throughout the course of the studies. Male Sprague Dawley rats weighing 250–300 g were provided by BioLASCO Taiwan Co., Ltd., Taipei, Taiwan. Rats were anesthetized, and then the ventral hair was shaved with an electric shaver without causing any damage to the skin. After sacrifice and excision, the rat skin was mounted on a modified Franz diffusion cell. The effective diffusion area provided in this setup was 3.46 cm^2^, and the recipient chamber was maintained at 37 °C using a circulating water bath system to maintain the skin temperature at 32 °C. Then, 20 mL of pH 7.4 phosphate buffer containing 20% ethanol was added to the compartment to maintain the conditions in the sink condition and stir at 600 rpm. Apply 1 mL each of the test preparation and the control group (5% linalool dissolved in ethanol) evenly on the skin surface, and then cover with paraffin film. At the predetermined time, withdraw 0.5 mL of the receiving fluid and add the same volume of fresh fluid to the receiving chamber. Quantify of the amount of linalool that penetrates the skin over time by HPLC [13,23,24]. 

At the end of the 24 h permeation study, the applied skin was carefully removed from the diffusion cell. Cotton was used to wipe off the residual drug on the surface of the smeared skin, it was washed with deionized water three times, and then shaken horizontally with receptor buffer overnight to extract the amount of linalool deposited in the skin. The resulting solution was filtered through a 0.45 mm membrane and analyzed for linalool content.

### 2.6. Chromatographic Condition

The modified HPLC method was used to determine the amount of linalool [13,23,24]. HPLC with a Hitachi L-7100 pump, L-5210/L-7200 autosampler, Hitachi L-4000H detector and Silversil^®^ C18 column with 250 mm × 4.6 mm id. and 5 μm particle size were used for the linalool analysis. The mobile phase consisted of distilled water and acetonitrile in a 6/4 ratio. The flow rate was 1.0 mL/min and the detection wavelength was 210 nm. For the analysis of linalool, a 50 µg/mL diazepam solution was used as an internal standard. The analytical method successfully demonstrated linearity (1–100 μg/mL) with a coefficient of determination (r) of 0.9992, a coefficient of variation of 7.23% and a relative error of 2.63%.

### 2.7. Skin Irritation Evaluation 

Male Sprague Dawley rats weighing 250–300 g were anesthetized by an intraperitoneal injection of 0.75 g/kg polyurethane aqueous solution. Then, 500 μL of distilled water (negative control), 0.8% paraformaldehyde (standard stimulation group), linalool solution and a tested linalool-loaded microemulsion formulation were evenly applied to 2.54 cm^2^ of shaved abdominal skin, and a paraffin film was sealed [25,26]. After 24 h, the animals were sacrificed and the treated skin was excised for histological examination. Briefly, the skin tissues were fixed in 10% formalin for at least 24 h. After fixation, the skin samples were rinsed with running water, dehydrated with a series of graded ethanol solutions and embedded in paraffin. Tissue blocks were cut into 10 μm thick sections, rehydrated and stained with hematoxylin and eosin (H&E), and the stained slides were inspected under a light microscope (Nikon Eclipse Ci, Tokyo, Japan) and assessed for histopathological changes associated with different formulations exposure.

### 2.8. Stability Study

The physical stability, namely the thermodynamic stability of the optimal formulation, was tested by the centrifugation method at 5000 rpm for 30 min and three cycles of a freeze–thawing cycle at temperatures between −21 °C and 25 °C with a storage time not less than 48 h at each temperature [27]. Appearance changes such as turbidity, phase separation, creaming or cracking of the tested linalool-loaded formulation were perceived and recorded.

The final optimal linalool-loaded formulation was stored in an amber glass bottle at 25 ± 2 °C and 60 ± 5% RH for three months. Samples were taken periodically for appearance and drug content analysis.

### 2.9. Data Analysis

The cumulative amount of linalool permeated was plotted as a function of time, and a linear regression analysis was used to measure the permeation rate (flux, μg/(cm^2^ h) of the drug. The lag time (LT, h) was the first detected time. The flux, linalool deposition in the skin over time (D_24h_) and LT were used to evaluate the permeation enhancement effect of microemulsion formulations. The data are stated as the mean ± standard deviation of three determinations. An ANOVA analysis followed by Tukey’s multiple comparisons using Winks SDA 6.0 software was used for testing the differences between the experimental formulations, and a *p*-value < 0.05 was considered significant.

A polynomial mathematical equation model with a response surface methodology provided by the Design-Expert software was used to clarify the relationship between the independent variables and the response, according to model *p*-value, lack-of-fit *p*-value, multiple correlation coefficient (R^2^), adjusted multiple correlation coefficient (adjusted R^2^), prediction residual sum of squares (PRESS). The *p*-values for model and lack of fit should be less than 0.05 and greater than 0.05, respectively. The coefficient of the X term represents the influence strength of the independent variable [25,26,28,29].

## 3. Results and Discussion

### 3.1. Phase Studies

**Figure 1:**
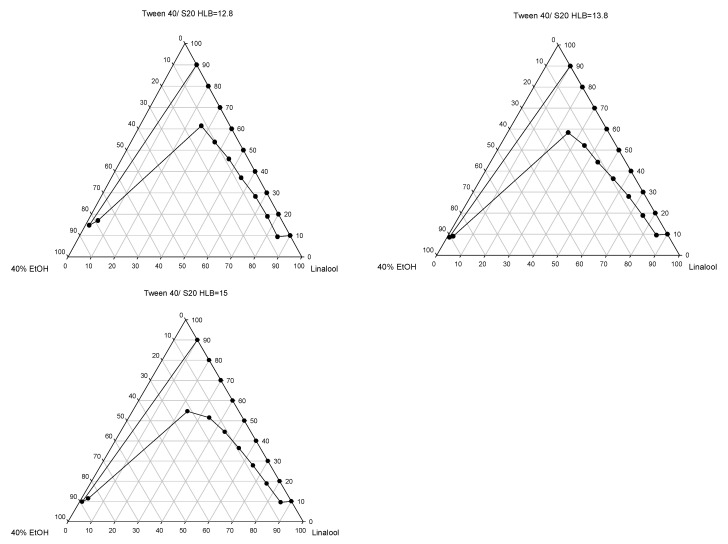
The pseudoternary phase diagrams of the oil/40% ethanol/mixture surfactant with different HLB values.

Mixing of the components in appropriate ratios is important for microemulsion formation [30]. The construction of the pseudoternary phase diagrams is a useful and important method for determining the appropriate composition of microemulsion formulations [31]; additionally, the HLB value and amount of surfactants in the microemulsions are the main factor that determine the formation of stable microemulsions and the enhancement permeation effect of the drug [32,33]. Accordingly, the pseudoternary phase diagram was constructed for different HLB values of mixed surfactants (Tween 40/Span 20), and as shown in Figure 1, the construction microemulsion area slightly increased via an increase in the HLB value of the mixture surfactants. The results may be attributed to the required HLB value of the oil. However, the ranges of components for the preparation of the model linalool microemulsions were selected from the pseudoternary phase with HLB 15 and then subject to a physicochemical properties analysis and in vitro permeation study.

### 3.2. Physicochemical Characteristics

The viscosity and average globule size of all the linalool-loaded microemulsions are measured and presented in Table 2. The viscosity ranged from 10.93 to 48.33 mPa·s, showing that the microemulsions possessed low viscosity. The ideal viscosity of the formulation varies by availability. However, formulations with a viscosity of approximately 10^6^ mPa·s may be suitable for topical applications. The average globule sizes ranged from 19.5 to 235.8 nm as for a nanoscale. Previous studies have reported that only particle sizes between 50 to 500 nm have the potential to penetrate the skin [34], indicating that the current design microemulsions might be well suited for dermal/transdermal delivery.

**Figure 2:**
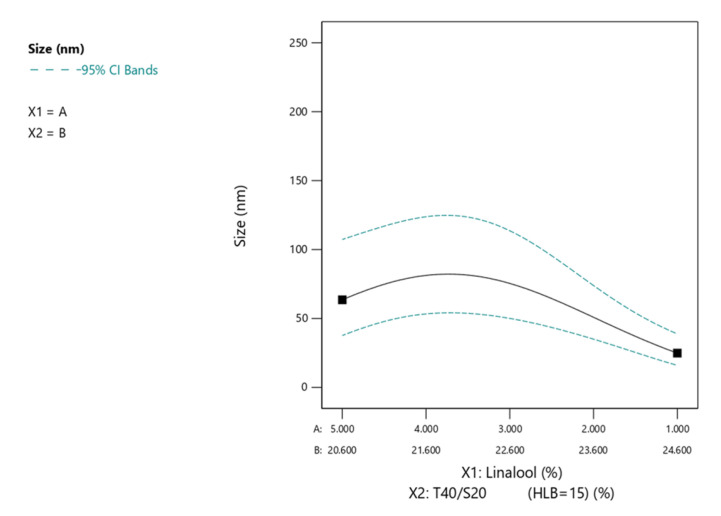
The two-component mixed plot of droplet size representing the influence of dependent variables on the responses. The dependent variable was fixed at cosurfactant phase (X_3_) of 0.33 and aqueous phase (X_4_) of 0.45.

To determine the influenced degree of component, the powerful statistical method response surface methodology was used [26,27,28]. The obtained data (independent variables and desired responses) were fitted to linear, interactive, quadratic and cubic equations. The result of the multiple regression analysis is represented in Table 3. It was found that droplet size showed a good relationship with the independent variables (Table 3). No suitable mathematical model for viscosity was found, probably because most microemulsions have a low viscosity. Among the main variables, X_1_, X_2_ and their interaction X_1_X_2_ showed a higher influence for droplet size. The response surface plot was generated for the responses (dependent variables) using the Design-Expert software to determine the impact of independent variables on dependent variables (Figure 2). It was found that smaller droplets could be formed when the drug concentrations of X_1_ were smaller and the surfactant concentrations of X_2_ were larger when X_3_ (cosurfactant) and X_4_ (distilled water) were fixed at a median value. Generally, the droplets of microemulsions increased in size as the concentration of the drug increased [35]. The average size of the microemulsions diminished with an increased amount of surfactant as a result of a larger oil–water interface [35,36].

### 3.3. In Vitro Permeation Study

**Figure 3:**
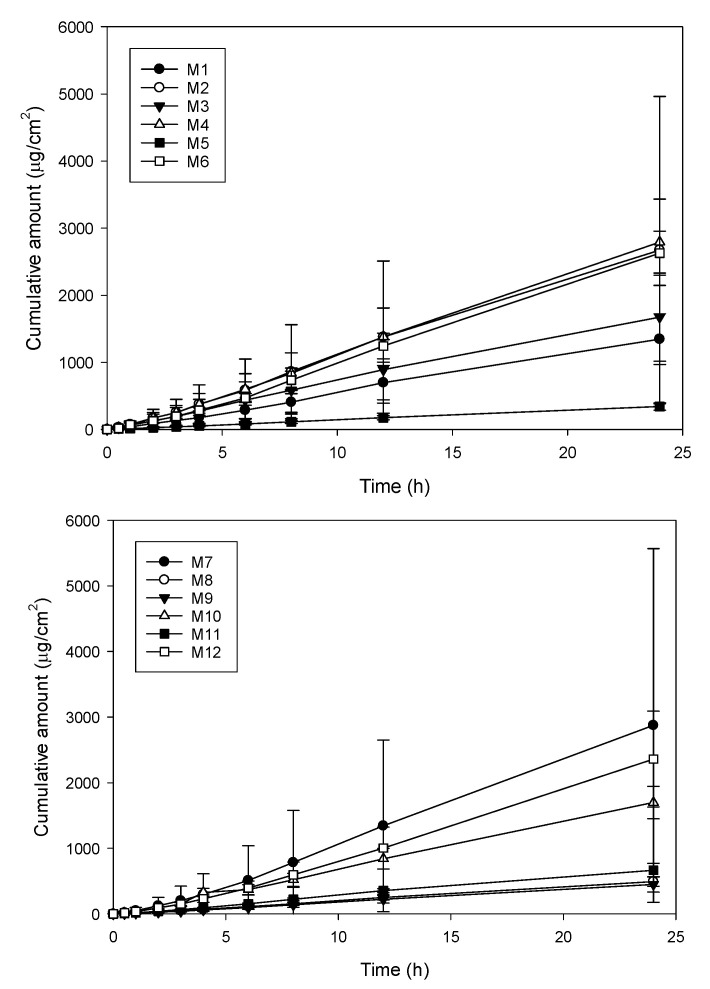
The permeation profiles of model linalool-loaded microemulsion formulations.

**Figure 4:**
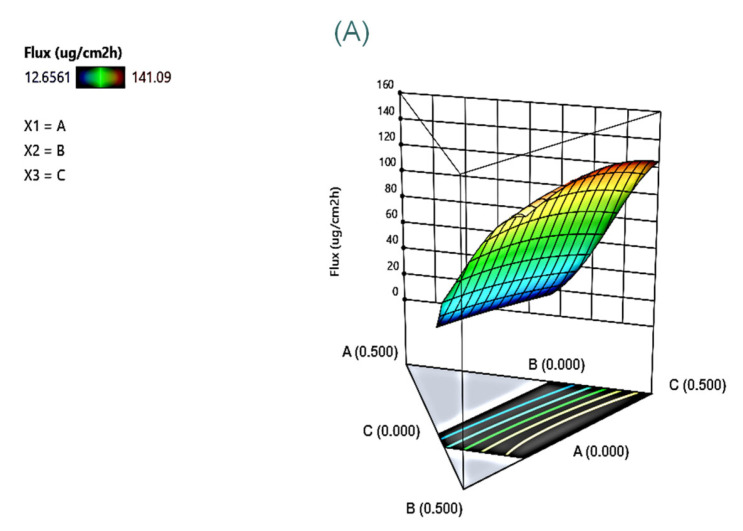

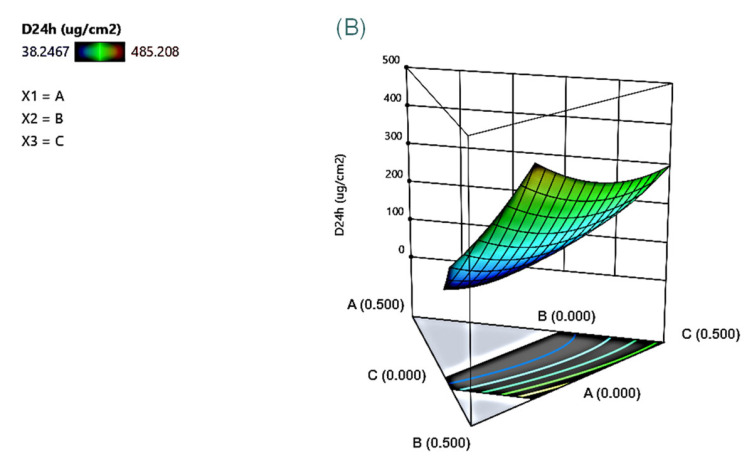
The response surface plots showing the effect of independent variables on the responses including flux (**A**) and deposition amount in skin (D_24h_, (**B**)) after 24 h application of formulations. The independent variable of aqueous phase (X_4_) was fixed at 0.5.

Figure 3 shows the permeation curves of these model formulations through the skin. The permeation curves indicated that drug permeation followed zero-order kinetics (R > 0.957). The permeation rate (flux) was calculated by a linear regression, with the permeation parameters of these model formulations listed in Table 2. The flux and D_24h_ ranged from 21.08 ± 2.48 to 121.79 ± 18.63 μg/cm^2^ h and 41.2 ± 4.0 to 443.4 ± 53.5 μg/cm^2^, respectively. The flux and D_24h_ of most linalool-loaded microemulsions were significantly higher than that of the control group of 5% linalool dissolved in 95% ethanol (18.71 ± 15.93 μg/cm^2^ h and 72.54 ± 21.70 μg/cm^2^ h). Such wide deviations demonstrated that drug permeability was greatly affected by the proportion of composition. From Table 3, it can be seen that the interaction factor of X_1_X_2_ and the main factor of X_1_ had the greatest effect for flux. The three-dimensional response surface plot (Figure 4A) showed that microemulsions with a higher drug concentration and surfactant concentration, as well as the cosurfactant concentration at medium concentrations, had higher flux. Increasing the loading dose is an effective way to increase the flux of various active compounds; for example, surfactants and cosurfactants can reduce the interfacial tension in microemulsions, resulting in a more dynamic and flexible layer, while adding a cosurfactant to the microemulsion can reduce the amount of surfactant used to form the microemulsion [37]. Although, the addition of excessive amounts of surfactant–cosurfactant might decrease the thermodynamic activity of the therapeutic compounds in the microemulsion, resulting in a decrease in the permeation rate [38]. In terms of D_24h_, the main factors of X_1_ and X_2_ as well as the interaction factor of X_12_ produced the greatest effect (Table 3). Figure 4B shows that X_1_ at a higher level and X_2_ as well as X_3_ both at medium levels had a higher drug deposition amount.

The optimization process provided by the software was applied to obtain an optimal linalool-loaded microemulsion with a code selected for X_1_, X_2_, X_3_ and X_4_ being 0, 0.25, 0.50 and 0.25, respectively, and the predicted values were 103.13 nm, 130.15 µg/cm^2^ h and 356.4 µg/cm^2^ for droplet size, flux and D_24h_, respectively. The optimal linalool-loaded formulation was prepared using the predicted values of the independent variables, and relevant data were then obtained by repeating the studies. The observed data were 110.21 ± 4.15 nm, 146.10 ± 4.87 µg/cm^2^ h and 342.38 ± 76.66 µg/cm^2^, respectively, which were in good agreement with the predicted values, indicating that the response surface methodology with a four-variable mixture design was an effective statistical skill for pharmaceutical formulation development and could be applied in linalool-loaded microemulsions design.

### 3.4. Skin Irritation

**Figure 5:**
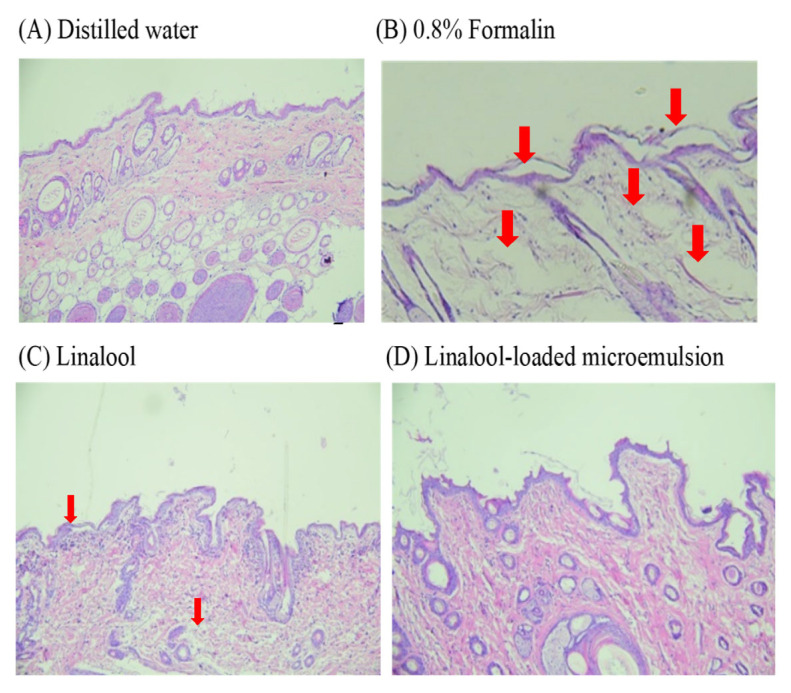
Photomicrographs of rat skin tissue after application of (**A**) negative control: distilled water; (**B**) positive control: 0.8% formalin; (**C**) linalool; and (**D**) linalool-loaded microemulsion. (Original magnification ×100). Arrows indicated change of position.

A skin histological examination was performed to evaluate the safety of linalool and the linalool-loaded formulation. The distilled water and 0.8% formalin solution treatment groups were used as the negative and positive (standard irritant) controls groups, respectively [26,39,40]. The photomicrographs of the negative control group showed clear epidermal and dermal layers (Figure 5A); in contrast, in the standard irritant group, the collagen fibers in the dermis were significantly swollen, the subcutaneous layer was severely edematous and the epidermal stratum corneum was slightly damaged and exfoliated (Figure 5B). For the linalool-treated group, there was slight swelling in the dermis layer demonstrating that linalool had some irritation for the skin (Figure 5C). There were no significant changes observed in the rat skin treated with the drug-loaded microemulsion formulation (Figure 5D) in comparison with the distilled water-treated skin (Figure 5A), suggesting an absence of any skin irritation. Similarly, previous studies have reported that microemulsion systems could reduce the irritation caused by active compounds [41,42].

### 3.5. Stability

The thermodynamic stability of linalool-loaded microemulsions was quickly tested by centrifugation at 5000 rpm for 30 min and three freeze–thawing (−21 °C and 25 °C) cycle tests. After these tests, the formulation did not show any phase separation or precipitation. There were also no significant changes in the droplet size or viscosity, except for a slight increase in the droplet size from 46.52 nm to 79.31 nm after the three freeze–thawing cycle tests (Table 4), indicating the excellent physical stability of the linalool-loaded microemulsions. Similarly, earlier studies have reported that microemulsion systems with a nanoscale droplet size and low interfacial tension between the water and oil phases make them thermodynamically stable [14,41,42].

After storage for 3 months at 25 ± 2 °C, 60 ± 5% RH, the appearance, viscosity and average droplet size of the linalool-loaded microemulsion formulation had no significant changes, and no drug was observed to crystallize. After three months of storage, the residual amounts of linalool were 103.12 ± 1.2%, indicating that the experimental preparations were quite stable.

## 4. Conclusions

The response surface methodology with a four-variable mixture design is a powerful statistical tool for drug dosage form development and was used in this study to realize the effect of independent variables (excipients proportion of a drug-loaded microemulsion formulation) on responses including the physicochemical characteristics of formulations and drug permeability and to obtain the optimal formulations. The viscosity and average globule size of all linalool-loaded microemulsions ranged from 19.5 to 235.8 nm and 10.93 to 48.33 mPa·s, respectively. The in vitro permeation study proved that the linalool permeability significantly increased by using the microemulsion as a delivery carrier. The optimized formulation of linalool-loaded microemulsions showed a significant increase in drug permeability through rat skin, including approximately 3.4-fold and 7.6-fold increases at Q_24h_ and D_24h_, respectively. The optimal microemulsion formulation showed thermodynamic stability via centrifugation at 5000 rpm for 30 min and three freeze–thawing (−21 °C and 25 °C) cycle tests. The tested linalool-loaded formulation was storage stable for at least three months at 25 °C, 60 ± 5% RH. In addition, the linalool-loaded microemulsion formulation showed less irritation compared to the standard irritant group (0.8% paraformaldehyde solution). The results of this study highlight that optimized linalool-loaded microemulsion formulations may be considered as promising vehicles for the topical application of linalool.

## Figures and Tables

**Table 1:** The composition of designed model drug-loaded microemulsion formulations.

Code Formulae	X_1_ 0~0.20	X_2_ 0~0.35	X_3_ 0~0.75	X_4_ 0~1.00
M1	0.108	0.000	0.325	0.566
M2	0.069	0.330	0.000	0.601
M3	0.112	0.195	0.689	0.004
M4	0.000	0.179	0.323	0.498
M5	0.200	0.000	0.750	0.050
M6	0.000	0.350	0.650	0.000
M7	0.000	0.000	0.750	0.250
M8	0.200	0.062	0.480	0.258
M9	0.200	0.106	0.000	0.694
M10	0.112	0.195	0.689	0.004
M11	0.200	0.350	0.362	0.088
M12	0.000	0.000	0.000	1.000

X_1_: Amount of linalool (1~5%); X_2_: amount of mixture surfactant (18~25%); X_3_: amount of 1,3-Propandiol (15~30%); X_4_: amount of aqueous water (40~60%); D_24h_: deposition amount in skin after 24 h application of formulation.

**Table 2:** The physical characterization and permeation parameters of designed model drug-loaded microemulsion formulations.

Formulae	Size (nm)	Viscosity (mPa·s)	Flux (μg/cm^2^/h)	D_24h_ (μg/cm^2^)
M1	38.6 ± 0.1	41.60 ± 0.99	58.61 ± 10.83	149.4 ± 28.0
M2	48.9 ± 0.4	27.57 ± 0.21	100.30 ± 16.45	243.6 ± 18.0
M3	38.8 ± 0.3	25.33 ± 0.39	68.37 ± 17.45	159.9 ± 23.7
M4	57.3 ± 0.6	36.93 ± 0.53	121.79 ± 18.63	313.3 ± 21.9
M5	19.5 ± 0.2	26.73 ± 0.97	14.34 ± 1.71	60.9 ± 33.4
M6	36.0 ± 0.1	18.93 ± 0.66	119.18 ± 11.44	443.4 ± 53.5
M7	92.2 ± 4.9	41.37 ± 1.11	119.78 ± 7.88	299.9 ± 24.5
M8	35.2 ± 0.8	20.30 ± 0.22	21.08 ± 2.48	45.7 ± 4.0
M9	75.5 ± 2.1	24.33 ± 1.26	19.57 ± 4.12	41.2 ± 5.9
M10	235.8 ± 6.7	17.83 ± 1.18	73.74 ± 7.72	112.5 ± 58.1
M11	26.9 ± 0.4	10.93 ± 0.39	28.02 ± 4.93	92.6 ± 56.2
M12	151.1 ± 1.3	48.33 ± 0.40	109.69 ± 28.35	294.3 ± 87.0

**Table 3:** The result of the statistical analysis of dependent variables and desired responses.

	Ln (Flux)	D_24h_	Ln (Size)
Regression Coefficient	Coefficient Estimate	Coefficient Estimate	Coefficient Estimate
b_1_ (X_1_)	−35.75	1770.79	−17.96
b_2_ (X_2_)	3.24	1595.15	−13.28
b_3_ (X_3_)	4.67	296.99	5.39
b_4_ (X_4_)	4.64	294.25	4.95
b_12_ (X_1_X_2_)	48.52	−6269.82	65.17
b_13_ (X_1_X_3_)	38.13	−3333.48	15.63
b_14_ (X_1_X_4_)	38.67	−3122.24	21.96
b_23_ (X_2_X_3_)	2.62	−1353.82	20.76
b_23_ (X_2_X_4_)	2.35	−1561.60	20.48
b_34_ (X_3_X_4_)	0.51	18.79	−4.31
Model (*p* value)	<0.0001	<0.0001	<0.001
R-Squared	0.9460	0.9257	0.6953
Adj R-Squared	0.9273	0.8999	0.5898
Lack of Fit (*p* value)	0.2259	0.9832	0.1917

Ln: Nature log; D_24h_: linalool deposition amount in skin after 24 h application.

**Table 4:** The viscosity and droplet size of a tested linalool-loaded microemulsion formulation before and after thermodynamic stability tests.

	Droplet Size nm	Viscosity mPa·s
Before test	46.52 ± 1.23	24.07 ± 0.43
Centrifugation test	43.70 ± 0.33	23.36 ± 0.44
Freeze–thawing cycle test	79.31 ± 5.82	25.02 ± 1.11
